# The Calibration of Displacement Sensors

**DOI:** 10.3390/s20030584

**Published:** 2020-01-21

**Authors:** Han Haitjema

**Affiliations:** Department of Mechanical Engineering, KU Leuven, 3001 Leuven, Belgium; han.haitjema@kuleuven.be; Tel.: +32-16374283

**Keywords:** displacement, sensor, calibration, interferometry

## Abstract

Displacement measuring sensors play an essential role in all aspects of dimensional metrology. They can be used for direct displacement measurements but more often they are part of a measurement system, such as an atomic force microscope, roughness tester or a coordinate measuring machine (CMM). In order to achieve traceable measurements that can be related to the meter, these sensors must be calibrated against a reference standard that is more noise- and error-free than the sensor under test. A description of the various methods to achieve the ultimate traceability, repeatability and accuracy of such a calibration system is the main part of this paper. Various interferometric methods will be reviewed including several methods that use directly a primary standard as a reference: either an iodine-stabilized laser or a frequency comb. It is shown that various methods exist to quantify or mitigate the periodic errors that are inherent to interferometric methods. Also it is shown that knowledge of this periodicity may lead to a separation of periodic and non-periodic non-linearity errors of both the calibration instrument as the sensor under test. This review is limited to small-range sensors, typically with a range <100 μm. It is concluded that today’s technology enables sound and traceable sensor calibration up to the sub-nano and even picometer level of uncertainties

## 1. Introduction

Displacement measuring sensors play an essential role in all aspects of dimensional metrology. They can be used for direct displacement measurements but more often they are part of a measurement system, such as an atomic force microscope or a coordinate measuring machine (CMM). Also they can be used as probe in surface topography or roundness measurements. In order to achieve traceable measurement that can be related to the meter, these sensors must be calibrated against a reference standard that is more noise- and error-free than the sensor under test. This review deals with the calibration of sensors with a range in the sub-mm range with uncertainties in the nm-level or smaller. Several calibration principles will be reviewed and compared. The calibration method can be quasi-static as well as dynamic. The probe can as well be basically static in its sensing direction, but be displaced lateral to this direction in order to measure a topography. The probe principles will be outlined in Section 2. Section 3 gives some common aspects of sensor calibration, independent of the senor principle or the calibration method. Section 4 reviews the major calibration principles found in the literature: laser interferometry, various applications of Fabry–Pérot cavities and X-ray interferometry. Section 5 reviews the extension to dynamic calibration systems.

## 2. Sensors to Be Calibrated

Dimensional metrology sensors that need calibration are basically capacitive and inductive sensors used in a variety of applications. An overview of the types and principles can be found e.g., in Doebelin [1] and Weckenmann [2], and together with some calibration strategies by Leach [3]. A short overview of the main types is given here:

- Inductive sensors, also called linear variable differential transformers (LVDTs). Inductive sensors are basically contactless, though the common embodiment is a probe that can move straight in/out of its shaft, while the position is determined by an internal electronic circuit [4]. A general characteristic is that the resolution can be very high, up to the sub-nm level, but this implies a high amplification that limits the range; for sub-nm resolution the range may be limited to ±10 μm. Inductive sensors are used both stand-alone as well as in roughness- and roundness testers.
- Capacitive sensors. Capacitive sensors utilize the physical fact that the capacitance between two electrodes depends on the geometry and the distance between these electrodes. Like inductive sensors, the sensitivity can be very high but for very high sensitivity the range is limited. Some types need two electrodes, others use the inductive effect when approaching a conductive medium so they can be principally contactless [5].
- Confocal sensor. This sensor uses a lens to focus a light beam on a surface, and it gives a focus error signal once the light is de-focused. The range and resolution depend on the configuration, but resolutions of nm-order can be achieved [6]. The most common application is as detection element in a CD or DVD player.
- Confocal chromatic probe. This sensor works similar to a confocal sensor, but the lens is chromatic on purpose and the spectrum of the reflected light is detected. From this spectrum the position of the reflecting surface is calculated. This has the advantage that directly a position is obtained depending on what color is in focus [7]. Depending on the configuration the resolution can be in the nm-range [8].
- Linear encoder. A linear encoder consist of a line scale that is read out opto-electronically. Important characteristics are the pitch and the interpolation quality of the read-out system. For high-resolution read-out the diffraction properties of periodic grids are used. Due to these diffraction properties the smallest useful pitch is about 0.4 μm. Interpolation errors will re-appear periodically with each displacement over an integer amount of pitch distances. Because of the scale the linearity can be very good over long distances, up to meters. In general the calibration characteristics will consist of the pitch deviations that may appear on a longer scale, with interpolations errors between the lines that repeat every pitch length. An overview of the technologies and the configurations is given by Cosijns [9]. When applied in probes covering small ranges these sensors may look like an inductive sensor though the working principle is basically different.
- Laser interferometer. The basic instrument that is used to achieve high accuracy, linearity and traceability is the laser interferometer that is on the market since the early 1980s [10]. An extensive overview of its applications is given elsewhere [11]. This instrument can be subject to calibration for small displacement, however it can very well act as the reference for calibrating other sensors. This is because the laser interferometer can be referenced to a primary standard in a single or in a few steps so that the traceability to the meter can be guaranteed [12]. For small displacements, the influence of frequency stability vanishes and the remaining factors that limit the uncertainty are noise, periodic non-linearity [13] and environmental factors, especially temperatures, that may affect the calibration set-up.

## 3. Common Aspects of Displacement Sensor Calibration Systems

### 3.1. Basic Calibration Set-Ups

**Figure 1:**
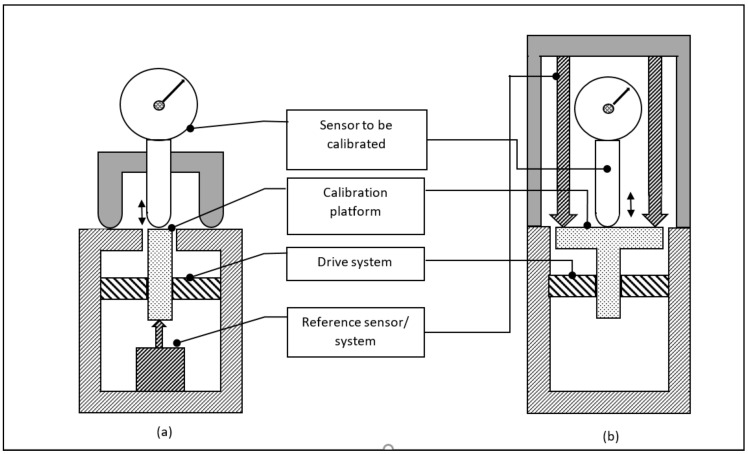
Common calibration set-ups for displacement sensor calibration. In the left-hand side (**a**) the reference sensor detects the displacement of the calibration platform from the other side as the senor to be calibrated, in the right-hand side (**b**) the reference sensor and the sensor to be calibrated detect the displacement of the calibration platform from the same side.

Two basic calibration set-ups were given by the author [14] and are depicted in Figure 1. The sensor to be calibrated, i.e., the sensor under test, is mounted on a calibration platform. This platform can be moved over the desired calibration range using a driving mechanism; for small displacements this is typically a piezo-transducer or a voice-coil. Once this sensor is moved, its detected displacement is recorded. At the same time the displacement of the reference sensor is read-out. Commonly this reference sensor is located at one side of the platform while the sensor under test measures from the other side (see Figure 1a), however sometimes both the reference sensor and the sensor under test measure the same surface from the same side (see Figure 1b). The displacement- and reference sensor can be oriented horizontally as well. Care must be taken to ascertain a straight and parallel movement of the calibration platform to avoid Abbe and cosine errors. When the probe moves over the surface while it is calibrated, the surface flatness is relevant. In that case also the movement must be strictly normal to the surface to avoid aligning problems and cosine errors.

The sensor under test can be any of the sensors discussed in Section 2 as well as other types of sensors.

### 3.2. Record and Display of Calibration Results

**Figure 2:**
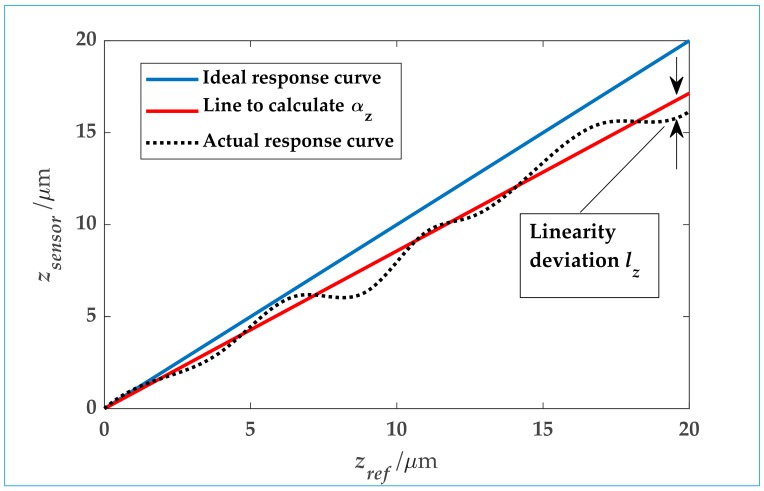
Example of a response curve and the derivation of amplification factor and linearity deviation.

The calibration results can be recorded in a table or a graph that displays the *z*-displacement as it is measured by the sensor to be calibrated against the *z*-displacement as it is measured by the reference sensor. In some fields it is common to distinguish between the amplification coefficient α_z_ (also called calibration coefficient) and the linearity deviation *l_z_*, that is the deviation of the sensor signal from a linear relation between the sensor to be calibrated and the reference sensor [15,16,17]. Figure 2 gives a graph as it is commonly displayed.

For sensors with a small deviation from linearity it can be more useful to display the deviations as a function of the reference value as in that case the calibration curve shows essentially a straight line without visible deviations. If measurements in a large displacement range are taken with smaller and larger steps, it may be useful to use logarithmic scales for either axes. For more formal reports such as calibration certificates a table of values and deviations, accompanied with an uncertainty estimate is most useful.

## 4. Displacement Sensor Calibration Principles

The sensor principles that can be used for a calibration system are basically listed in Section 2, however for a reference sensor the resolution, inherent calibration possibilities, and, if possible, a direct traceability to a primary standard, are basic properties. A short overview was given by Leach [3]. This section gives an overview of principles and systems used in practice.

### 4.1. Systems Based on Displacement Laser Interferometer

**Figure 3:**
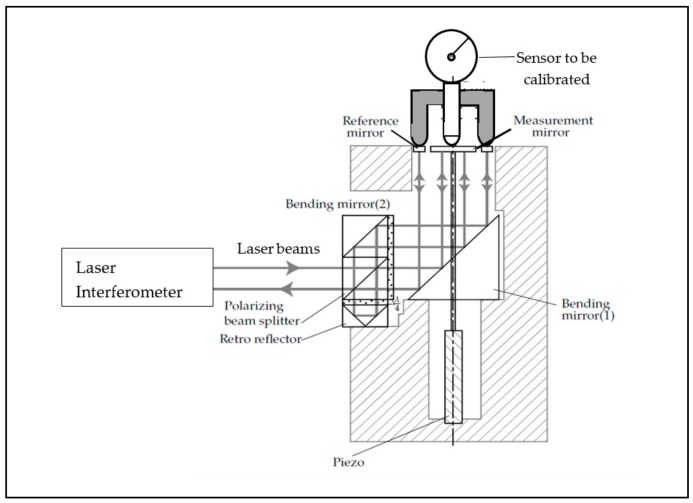
Principle of a sensor calibration system based on a plane mirror laser interferometer. Courtesy of S. Cosijns.

Laser interferometers that are basically Michelson-type interferometers are known for their high accuracy and versatility, combined with a possibility of dynamic measurements [11]. Figure 3 shows a calibration system based on a laser interferometer system that was used in order to calibrate a 3-D probe in several directions [18]. The double-path interferometer design aims to minimize drift effects in the set-up.

**Figure 4:**
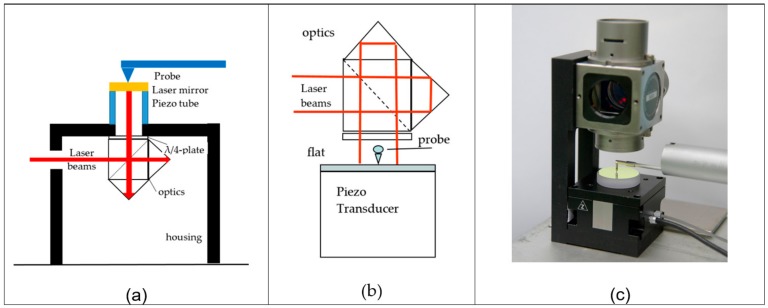
Dynamic calibration set-ups based on a laser interferometers as a reference sensor. A laser mirror is moved by (**a**) piezo transducer and measured simultaneously by a probe and the laser interferometer. (**b**) The same principle as (**a**). however the mirror movement is measured from the same side. (**c**) Picture of a set-up as sketched in (**b**).

For a dynamic calibration the drift may be less important than the linearity deviation and/or the dependence on the frequency because only the (sinusoidal) varying part of the signal is considered; therefore less-complicated set-ups compared to the set-up shown in Figure 3 have been designed. Figure 4 shows the schematic of such set-ups. Figure 4b shows a set-up where a flat mirror is measured simultaneously by an LVDT sensor from a roughness- or roundness measuring instrument, while as a reference sensor a laser interferometer in a flat-mirror configuration is used, that reduces the Abbe-errors that become relevant when the mirror does not have a parallel movement.

**Figure 5:**
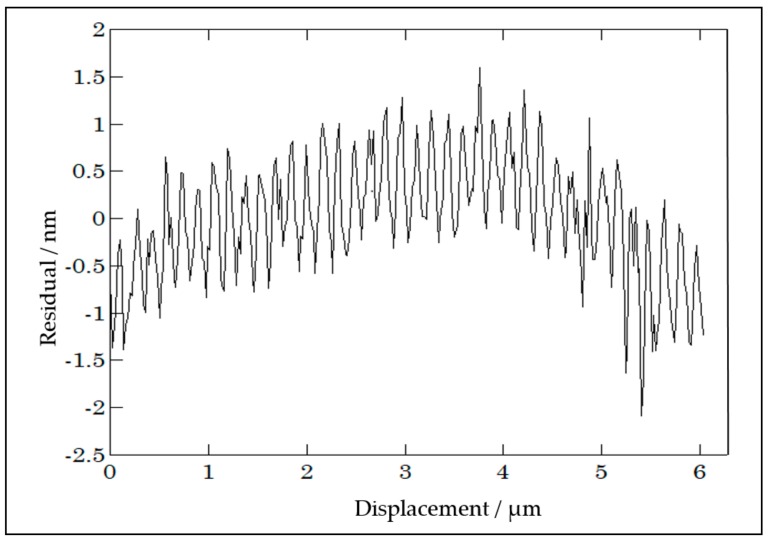
Calibration result of an inductive probe (Mahr Millitron), using the calibration set-up as sketched in Figure 3, displayed as the deviation from linearity (see Figure 2), after [18].

A disadvantage of laser interferometers is their remaining non-linearity that repeats every half-wavelength of displacement. These are commonly in the sub-nm region but they can amount to several nanometers for sub-optimal aligned systems. This makes calibration set-ups based on laser interferometer systems less appropriate for the calibration of laser interferometer systems that exhibit a similar type of linearity deviations. However the characteristics of other probe types, where there is no reason to assume that these exhibit periodic deviations with the same periodicity as the reference laser interferometer, can be distinguished from the laser interferometer non-linearity by a Fourier analysis of the calibration result. As an example, Figure 5 shows a calibration characteristics of a capacitive probe that is calibrated by a laser interferometer system. The periodic deviation with 0.15 µm wavelength (≈λ/8) and about 0.5 nm amplitude that originates from the reference laser interferometer can clearly be distinguished from the longer-range deviation of the probe itself.

Sensor calibration systems based on laser interferometer systems are further described by Hermann [19,20,21], Jing [22], Zheng [23], Zhang [24], Ge [25], and Li [26]. These all are based on the principles discussed in this section. The interaction of the periodic laser interferometer non-linearities with the probe under calibration was further studied by Köning [27] and Sacconi [28]. Also, dedicated laser interferometers been designed that exhibit hardly or no detectable non-linearities [29,30], or systems were designed to compensate these [31,32,33,34]. In that case these may compete for the noise-characteristics with the systems based on Fabry–Pérot interferometers as discussed in the next section. 

### 4.2. Systems Based on a Fabry–Pérot Interferometer

The major difference of a Fabry–Pérot interferometer with a Michelson- or Fizeau interferometer is that very sharp interference peaks appear at half-wavelength distances. This characteristic has the disadvantage over Michelson-type interferometers (see Section 4.1) that in-between these interference peaks no useful calibration is possible. On the other hand this enables the stabilization of tunable lasers on such a cavity; where a small displacement of one cavity mirror gives a major change in the frequency of the laser that is stabilized on his cavity.

In the first place a primary length standard, an iodine-stabilized laser, is based on a Fabry–Pérot cavity, where the laser frequency is defined once it is stabilized on an iodine absorption dip [35]. This means that a stabilization on this same dip appears at exactly each λ/2, where λ is the wavelength, displacement of one of the cavity mirrors. In the second place a Fabry–Pérot interferometer can be used directly as a laser cavity where the laser frequency is directly related to the cavity length and can be determined by a beat-measurement against a primary standard. In the third place a Fabry–Pérot cavity can act as an external cavity on which another laser can be stabilized; where the frequency of this laser is calibrated against a reference laser; e.g., a primary length standard. These possibilities are described in Section 4.2.1, Section 4.2.2, Section 4.2.3 and Section 4.2.4.

#### 4.2.1. Varying the Cavity Length of a Primary Length Standard as a Displacement Calibration Device

**Figure 6:**
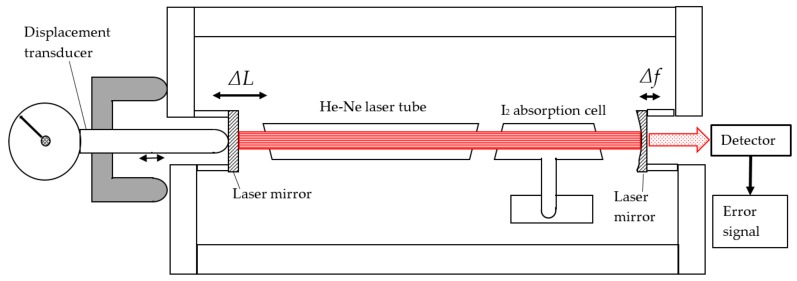
Sketch of an iodine-stabilized laser when used as a displacement calibration device.

The use of an iodine-stabilized laser as a displacement calibration device was first explored by Ottmann and Sommer [36]. They displaced one mirror of the cavity of an iodine-stabilized laser using a micrometer and took the micrometer position where an iodine absorption peak was observed as a calibration position for the micrometer. Where the micrometer-resolution is at best a fraction of a micrometer, and the effective laser mirror displacement had sub-nm resolution, this was rather an overkill for the application, but the principle was new. A further elaboration based on this principle was carried out by Brand and Herrmann [37]. Small displacements of one laser mirror generate a laser frequency change. By recording the 3f-signal zero crossings of the iodine hyperfine structure components, the displacement can be derived. As the frequency differences of the iodine R(127) 11–5 transitions are known within a standard uncertainty of 5 kHz [35], the mirror position can be derived with an uncertainty of 4 pm. Within the λ/2 repeating range, steps of nominally 10, 18, 84, and 165 nm could be made over a total calibration range of 13 µm. A schematic of this set-up is given in Figure 6. A major advantage of this method is the very direct traceability loop: as a primary standard is directly used the traceability is directly guaranteed. Despite this advantage this method has not become widespread because of the high demands to the straight and parallel movement of the laser mirror, the discontinuous calibration points and the heat generated by the laser.

#### 4.2.2. Varying the Cavity Length of a He–Ne Laser: The Measuring Laser

**Figure 7:**
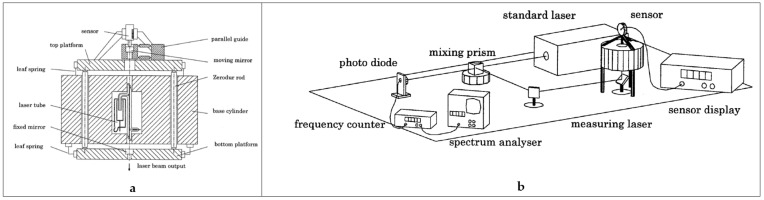
Schematic cross section of a He–Ne measuring laser (**a**) and overview of the calibration set-up (**b**). Courtesy of S. Wetzels.

A major disadvantage of the measuring laser based on an absorption cell is the discontinuous character: the cavity length needs to be carefully tuned on a molecular absorption line for every calibration point. This disadvantage has been overcome by the measuring laser based on a He–Ne laser where one mirror is used as the calibration platform. The cavity has to be long enough for the He–Ne to emit laser light, on the other hand it must be short enough to make the laser operate in single mode. This limits the cavity length to the 100–150 mm range. The working range of about 1500 MHz enables a frequency calibration of the laser by a beat-measurement with an iodine-stabilized laser. Figure 7 shows the system as it was realized by Wetzels and Schellekens [38,39]. Figure 7a gives the details of the measuring laser design; Figure 7b gives the set-up where it is used for calibration. The displacement Δ*L* is measured as a frequency shift Δ*f* and is in good approximation given by:

(1) $$\Delta L=\frac{1}{n_{air}}\left(\frac{\Delta f}{f}L^{*}+m\frac{c}{2f}\right),$$

where *n_air_* is the air refractive index, *f* is the absolute frequency, L^*^ is the optical cavity length, *c* is the light velocity and *m* is an integer number that applies when a measurement is made over multiple steps of the half wavelength λ/2 where mode-jumps occur. The laser frequency can be derived from a beat-measurement with a primary length standard: an iodine-stabilized laser with a typical standard deviation of 50 kHz, that corresponds to a displacement of 0.012 nm in this design.

Compared to the system described in Section 4.2.1, this set-up has the advantage of a continuous and instantaneous measurement: no control loop is needed to adjust a distance to enable calibration; the measurement as indicated by the frequency counter can directly be transferred to a displacement using (1). Figure 7a also indicates that quite some thermal mass is included in the set-up in order to reduce the effect of heat that is generated by the He–Ne discharge tube. However the uncertainty was still large due to varying temperature gradients in the system. This limited the uncertainty to some 25 nm, despite the resolution of 0.012 nm [39]. A similar system was developed by Çip [40] who used it to determine the non-linearity of laser interferometer systems in an optical configuration like in Figure 3.

#### 4.2.3. Using an External Cavity and a Slave-Laser: The Metrological Fabry–Pérot Cavity

**Figure 8:**
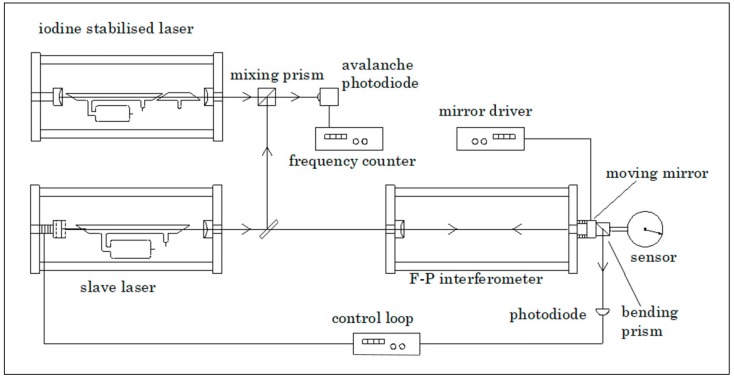
Overview of the metrological Fabry–Pérot interferometer set-up. Courtesy of S. Wetzels.

The Ne–Ne laser tube as a heat source can be removed from the cavity and the laser frequency of another He–Ne laser can be stabilized on this same cavity. This takes away the temperature/heating problem at the expense of another laser and a feed-back system for stabilizing an external laser on the cavity. For the application of a He–Ne laser in a 1500 MHz frequency range, this cavity still needs to be relatively long, typically 140 mm. To minimize temperature effects, the cavity length is stabilized using zerodur rods and the effect of the air refractive index is reduced by the placement of a vacuum tube in the cavity. Figure 8 gives a schematic of such a set-up [39,41].

The slave-laser frequency must be modulated in order to find the appropriate intensity-maximum. This set-up was used to calibrate various displacement sensors, also it was used for the determination of periodic deviations of laser interferometer systems [42,43,44,45]. Riis [46] used a similar set-up to calibrate the characteristics of piezo-elements.

In this set-up there is a relationship between the range in which a He–Ne laser can operate (about 1000 MHz), the frequency detection range of the avalanche diode and the frequency counter (about 1000 MHz as well) and the free spectral range (FSR) of the Fabry–Pérot cavity, i.e., the resonance frequency range for a displacement of λ/2 of the moving mirror. In general the free spectral range (FSR) is given by:

(2) $$FSR=\frac{c}{2nL},$$

where *c* is the speed of light, *n* is the air refractive index and *L* is the cavity length. The frequency range of the slave-laser, the detector frequency range as well as the Fabry–Pérot cavity length determine the range over which the moving mirror of the Fabry–Pérot cavity can be displaced before a discontinuity appears. Widening the frequency range, e.g., by using a diode laser with a wider tuning range as the slave laser, together with using a wider-range photodiode, enables a larger continuous measurement range as well as a shorter Fabry–Pérot cavity to be used. Based on this, Howard, Stone and Fu [47] developed a system based on a 50 mm Fabry–Pérot cavity and a 20 GHz bandwidth frequency counter system, obtaining a 2.1 µm continuous measurement range. This frequency detection range was further extended to a 65 GHz bandwidth by Bitou [48]. The high-frequency measurements needed are further enabled by the use of an optical frequency comb, e.g., in [49] where a 140 GHz bandwidth was achieved, using a 75 mm Fabry–Pérot cavity. In this work, a second cavity was used with a similar length, without moving elements, to monitor and compensate for changes in the air refractive index; as earlier developed by Schibli [50]. Similarly, Zhu et al. [51] achieved 0.46 pm resolution for a 50 mm cavity. Çip et al. [52] used a femtosecond comb spectrum to obtain a high bandwidth frequency measurement using a 65 mm Fabry–Pérot cavity. The achieved uncertainty was 22 pm over 650 nm displacement. A method to reduce the cavity length was proposed by Joo [53].

#### 4.2.4. Using a Sub-Mm Range Fabry–Pérot Cavity

**Figure 9:**
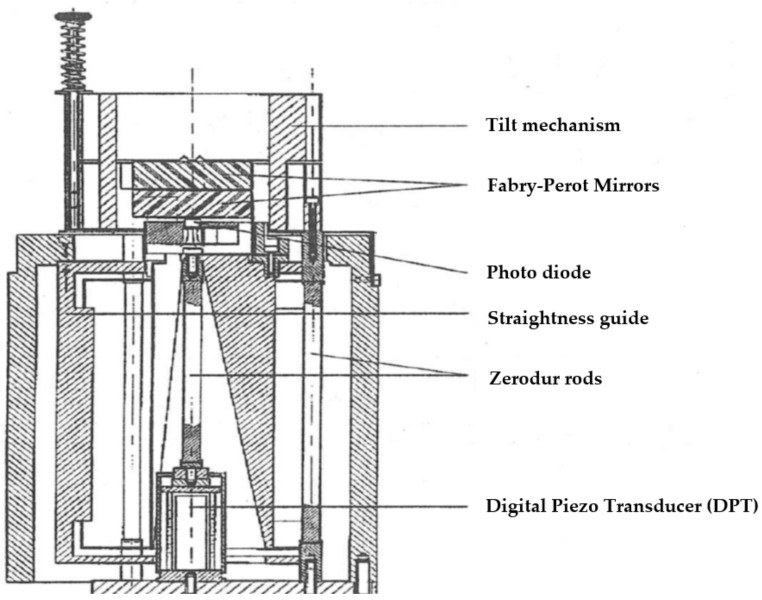
Sketch of a calibration system based on a digital piezo transducer, calibrated by a Fabry–Pérot interferometer.

When reducing the length of a Fabry–Pérot cavity, the influence of temperature effects and the air refractive index reduce proportionally. However, when the cavity length becomes <10 mm the common design with convex mirrors does not hold and plane mirrors must be used. This puts additional requirements to a parallel movement. The free spectral range becomes very large, which implies that lasers with a limited tuning range cannot be stabilized on an arbitrary cavity length. However, if calibrations in λ/2 intervals are sufficient, an accurate calibration may be obtained. The author developed such a system as a calibration option to a displacement generator based on a digital piezo transducer [54,55]. This transducer (Queensgate DPT-C-S) has an internal feedback to a capacitive sensor and achieves sub-nm resolution in a displacement range of 15 µm. Figure 9 gives a schematic of the set-up. The Fabry–Pérot cavity has a length of about 80 µm. The calibration of the set-up is performed by illuminating the Fabry–Pérot cavity from above by a laser; the photodetector detects whether a transmission peak is reached. This peak is detected using a lock-in amplifier while either the cavity is given a small periodic vibration (4 nm amplitude) or by giving a tunable diode laser a frequency modulation of 20 GHz. A calibration curve could be obtained with a repeatability of 0.1 nm, where the system hysteresis of about 1 nm was the major remaining uncertainty source. After removing the upper mirror and the tilt mechanism, the system was used to calibrate displacement sensors.

### 4.3. Systems Based on X-ray Interferometry

**Figure 10:**
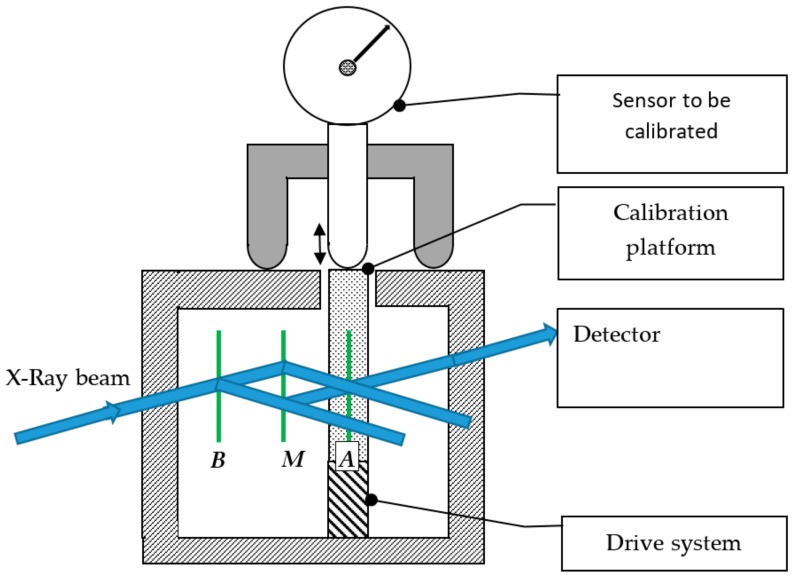
Simplified sketch of an X-ray interferometer system. Lamella are indicated as B, M and A.

The most accurate, but also the most elaborate, method to obtain small displacements with a high resolution is the X-ray interferometer. By its design, the fringe spacing in an X-ray interferometer is determined by the spacing of diffraction planes in the crystal. As the atomic lattice parameter of silicon (0.192 nm for the (220) planes) is well established in the literature it can be considered as a traceable standard of length [56], X-ray interferometry enables a traceable displacement measurement with a resolution within 0.2 nm [57]. A simplified sketch of the principle is given in Figure 10. X-rays are incident on the first lamella (*B*) at the Bragg angle, producing two diffracted beams. These two beams are incident on the second lamella (*M*). Two more pairs of diffracted beams are produced and one beam of each pair is incident on the third lamella (*A*). When the third lamella (*A*) is displaced, a Moiré fringe pattern is produced that causes an intensity at the detector that varies sinusoidally with the displacement with a period of one X-ray fringe (0.192 nm). In reality the interferometer is made from a single crystal of silicon and a strictly straight and parallel movement of the third lamella (*A*) is achieved by a flexure stage design. It is driven by a piezo stage and it has a range of 10 µm [57]. This range has been extended by combining this interferometer with a plane mirror interferometer, where the X-ray interferometer was used for the fraction determination within one fringe displacement of the interferometer [58,59]. Further it has been used to verify the performance of displacement measuring optical interferometers, where a detailed set of comparison measurements between the X-ray interferometer and a dual channel Fabry–Pérot optical interferometer (DFPI) have been made to demonstrate the capabilities of both instruments for picometer displacement metrology. The results show good agreement between the two instruments, although some minor differences of less than 5 pm have been observed [60]. An X-ray interferometer, combined with a laser interferometer, was used for the calibration of LVDT’s by Park [61].

## 5. Dynamic Calibration Platforms

**Figure 11:**
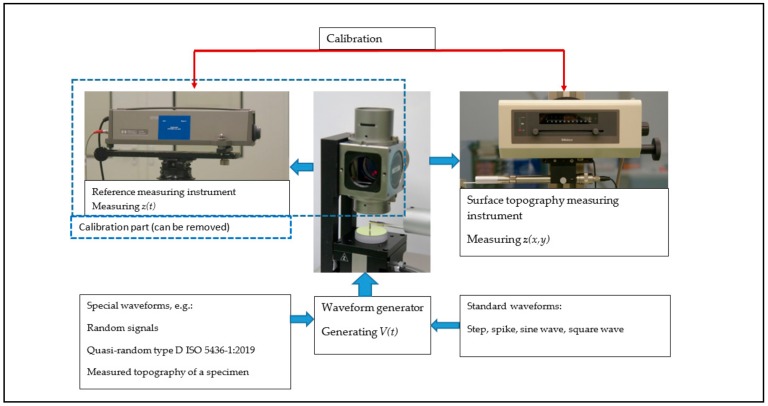
Scheme of a dynamic probe calibration set-up. The calibration part (indicated by a dotted line) can be removed after the system is calibrated.

Where all the systems discussed in Section 4 can perform a static calibration and determine a calibration characteristic for a probe similar as in Figure 2, many probes are not just used to measure a single displacement, but they are applied for measuring a profile or even a topography. In such a measurement the dynamic probe properties become relevant, as well as the probe size and geometry. Regarding the dynamic probe properties, these can be determined using the technologies and some of the set-ups discussed in chapter 4. For example, the calibration of the *z*-axis of a stand-alone Atomic Force Microscope (AFM), while measuring a flat surface that was being moved at calibrated displacements, was shown by three authors [39,47,55]. More traditionally, dynamic calibration systems, sometimes referred to as ‘moving tables’ were used in the calibration of roughness measurement instruments [62,63,64,65,66]. Figure 11 gives a general scheme of such a set-up. A time-dependent signal is fed to a displacement generator. This displacement is measured by the probe/instrument under calibration. For calibration purposes, this displacement can be measured simultaneously by a reference system. In most cases the generated waveform is a function of time, where the instrument to be calibrated measures the z-coordinate as a function of (*x,y*) position. In case of a sufficient constant lateral probe speed this gives no significant problems, however a direct link with an AFM controller has also been established [47]. This set-up is most appropriate to calibrate stand-alone probes or surface topography measuring instruments such as roughness and roundness testers.

Where the system depicted in Figure 11 has a rather macroscopic size and is less appropriate for e.g., AFM calibrations, miniaturized versions have been developed for AFM vertical [67] and lateral [68] calibrations. A portable system with integrated reference laser interferometer was developed by Pisani [69] as well as by Liang [70]. Next to the simulation of a step standard, a calibration based on sinusoidal signals is rather appropriate, as in enables a direct comparison to the surface parameter *Rq* or *Sq*. Also it enables a bandwidth check and the calibration of a proper filtering performance of the instrument [64,67]. A surface topography of a real object can be measured that is repeated as a simulated measurement using the calibration set-up. In this way the calibration comes close to a system check that is beyond just the probe displacement calibration.

## 6. Discussion

Many probe calibration systems have been developed that enable displacement measuring probes and instruments to be calibrated from the µm to the pm uncertainty range. Table 1 gives a summary of the characteristics of the main technologies used. This summary is schematic, the nuances are to be found in the preceding chapters and in the literature. These calibration systems are able to provide adequate traceability and uncertainty. However the application is still mainly inside the research- and national metrology institutes where these provide the traceability for reference instruments that calibrate the material reference standards (such as step height standards) that are easier to handle than the rather elaborate calibration systems. Some portable systems that enable dynamic calibration have been designed that can be used to calibrate sensors in the field. In due course, the commercially available laser interferometers have achieved nm and sub-nm uncertainty and the methods to detect or avoid non-linearities have developed to such an extent that the usefulness of Fabry–Pérot cavities may be questioned. On the other hand, the development of the Frequency comb and related high-frequency detection technologies have enabled Fabry–Pérot based technologies to achieve pm range uncertainties.

## Figures and Tables

**Table 1:** Schematic overview of major sensor calibration technologies.

	Piezo with L.I. ^1^ Readout	Piezo with Feed-back and L.I. Calibration	Iodine Stabilized He–Ne Laser	Measuring Laser	Fabry–Pérot Cavity	X-ray Interferometer
**Range**	10 µm	10 µm	13 µm	300 µm	100 µm	13 µm
**Resolution**	10 nm	1 nm	0.04 nm	0.012 nm	0.46 pm	2 pm
**Uncertainty**	20 nm	5 nm	1.4 nm	25 nm	1.2 nm	5 pm
**Bandwidth**	10 kHz	100 Hz	DC	10 Hz	DC	1 Hz
**Advantage**	Not complicated	Not complicated	Direct traceability	Accurate, traceable	Accurate, traceable	Highest accuracy
**Disadvantage**	L.I. needed	Indirect traceability	Impractical, discontinuous	Drift due to heat effects	Complicated set-up	Laborious, complicated
**Potential Application**	Separate probe, roughness tester, roundness tester	Separate probe, roughness tester, roundness tester, AFM	Separate probe	Separate probe, table-top AFM, L.I.	Separate probe, table-top AFM, L.I.	L.I. calibration, table-top AFM or STM, separate probe

^1^ L.I.—Laser Interferometer.
